# Advances in cryptococcal infections-associated immunopathology and potential therapeutic strategies

**DOI:** 10.3389/fphar.2026.1776489

**Published:** 2026-05-14

**Authors:** Nadezhda Sachivkina, Sarmistha Saha, Regina Gurina, Olga Kuznetsova, Natallia Zhabo, Marina Avdonina, Olga Pilshchikova

**Affiliations:** 1 Department of Microbiology V.S. Kiktenko, Institute of Medicine, Peoples’ Friendship University of Russia Named After Patrice Lumumba (RUDN University), Moscow, Russia; 2 Department of Biotechnology, Institute of Applied Sciences & Humanities, GLA University, Uttar Pradesh, Mathura, India; 3 Agrarian and Technological Institute, Peoples’ Friendship University of Russia Named After Patrice Lumumba (RUDN University), Moscow, Russia; 4 Department of Biochemistry T.T. Berezov, Institute of Medicine, Peoples’ Friendship University of Russia (RUDN University), Moscow, Russia; 5 Department of Foreign Languages, Institute of Medicine, Peoples’ Friendship University of Russia Named After Patrice Lumumba (RUDN University), Moscow, Russia; 6 Department of Linguistics and Intercultural Communication, Moscow State Linguistic University, Moscow, Russia; 7 Department of Therapeutic Dentistry, Institute of Medicine, Peoples’ Friendship University of Russia (RUDN University), Moscow, Russia

**Keywords:** antifungal resistance, antifungal therapy, cryptococcal meningitis, cryptococcus, drug repurposing, immunopathology, small molecule antifungals

## Abstract

Globally, the *Cryptococcus* species, particularly *Cryptococcus neoformans* and *Cryptococcus gattii,* have a significant impact on human health. Concurrent with the evolving epidemiology of cryptococcosis, it has now become evident how host immunity contributes to the pathophysiology of severe disease. The only pharmacological antifungal therapeutic agents available are 5-flucytosine, fluconazole, and amphotericin B. Echinocandins, the newest fungicidal class for invasive mycoses, are ineffective against cryptococcosis caused by *C*. *neoformans*. Antifungal treatment is hampered by the high toxicity, elevated resistance rate, and difficulty of currently known antifungal compounds crossing the blood–brain barrier, in addition to the limited pharmaceutical choices. Therefore, it is imperative to find novel therapeutic options for cryptococcal infections. This article will summarize what is now understood about the immunopathology linked to cryptococcal infections that produce a tolerant phenotype. We also outline the current possible antifungal therapeutic approaches to control cryptococcal illness. We go into further detail on the most recent developments in our knowledge of the adaptive and intrinsic resistance mechanisms used by *Cryptococcus* species to avoid medical interventions. Together with developments in genomics technology and high-throughput screening techniques, knowledge of the processes governing anti-cryptococcal drug resistance will, in general, spur innovation and quicken the discovery of antifungal drugs.

## 1 Introduction

The global burden of fungal disease has increased over time, encompassing both a rise in overall fungal infections including superficial and mucosal forms, and a concurrent increase in invasive fungal infections, particularly among immunocompromised populations (Brown et al., 2012; Sachivkina et al., 2023; Sachivkina et al., 2022). Four genera, including *Cryptococcus, Candida, Aspergillus*, and *Pneumocystis,* account for about 90% of all documented fungal-related fatalities (Brown et al., 2012). In addition to diagnostic delays, treating fungal infections is more challenging than treating bacterial or viral illnesses because of the similarities between eukaryotic fungi and humans. There are currently very few antifungal medications on the market, and their effectiveness is constrained by toxicity, a limited range of action, harmful drug interactions, resistance development, and, occasionally, excessive cost (Scorzoni et al., 2017). One of the common fungi that affect human health, the species complex of the basidiomycete *Cryptococcus neoformans* is particularly concerning. Although there are at least 37 species in the genus Cryptococcus, the two main species that cause potentially fatal cryptococcal meningitis are *C. neoformans* and *Cryptoccocus gattii*; immunocompromised hosts are more at risk, while infections with *Cryptococcus* have also been reported in immunocompetent hosts (Perfect et al., 2010; Kwon- Chung et al., 2014). Cryptococcal meningitis is a life-threatening disease and results from dissemination of the fungal pathogen *C*. *neoformans* from the lungs to the central nervous system (Betancourt et al., 2025). An estimated 223,100 cases of cryptococcal meningitis and 181,100 fatalities are reported worldwide each year (Rajasingham et al., 2017). Latent *C. neoformans* infection in the lungs is contained by structured aggregates of immune cells known as granulomas (Betancourt et al., 2025). Due to limited access to medications and the high expense of effective treatments, the majority of deaths are reported from nations with minimal resources, highlighting the urgent need to find reasonably priced medicines against these lethal fungal pathogens (Loyse et al., 2019).

*Cryptococcus neoformans* is classified as a class 2 pathogen because it has developed virulence traits that enable it to infect individuals with weakened immune systems. However, immune reconstitution inflammatory syndrome (IRIS)-associated cryptococcosis after HIV/AIDS treatment and *C. gattii* infections in healthy individuals suggest that cryptococci can affect both immunocompromised and immunocompetent individuals. While *C. gattii* prefers immunocompetent individuals, *C. neoformans* traditionally targets immunosuppressed populations, such as those with advanced HIV-AIDS, various T cell deficiencies, pregnancy, chronic lung, renal, or liver diseases, cancer, and patients undergoing immunosuppressive therapy (Chen and MeyerW, 2014).

Protection against cryptococcal infection is associated with effective Th1/Th17 adaptive immune responses and classical macrophage activation that promotes fungal clearance (Mukaremera and Nielsen, 2017; Campuzano and Wormley, 2018; Chen et al., 2005; Murd et al., 2014; Elsegeiny et al., 2018). While sterilizing immunity requires effective pathogen clearance mechanisms, these responses may involve inflammatory processes that can also contribute to host tissue damage. A finely controlled balance between Th1, Th17, and Th2 responses is really necessary for the optimal immune response to cryptococcal infection. This balance regulates fungal development while avoiding severe tissue damage and immunopathology (Mukaremera and Nielsen, 2017). The issue of IRIS is a vivid example of the harmful effects of increased inflammation during cryptococcal infection. Cryptococcal IRIS development is mostly linked to solid organ transplant recipients, HIV + patients, and pregnant women. It is brought on by the recovery of certain immune responses, which cause increased host inflammation and local organ damage (Haddow et al., 2010). In HIV + individuals, cryptococcal IRIS comes in two varieties, firstly, unmasking cryptococcal IRIS, which starts soon after ART initiation in patients without a prior diagnosis of cryptococcosis and may be its initial manifestation; and secondly, paradoxical cryptococcal IRIS, which happens after starting antiretroviral therapy (ART) and manifests as a worsening or recurrence of clinical symptoms in the same or new site even with successful antifungal therapy (Williamson et al., 2017; Bowen et al., 2016). Post-infectious inflammatory response syndrome (PIIRS), a paradoxical immune response linked to severe neurological illness, can also happen to non-HIV individuals with cryptococcal meningitis when immunosuppressive medication is reduced (Williamson, 2015).

Three forms of cryptococcal meningitis can be recognized. First, in HIV + patients not yet on ART, disease is characterized by a high fungal burden, with pathogen-mediated damage predominating; low Th1 cytokines (e.g., TNF-α, IFN-γ) suggest minimal immune-driven pathology (Jarvis et al., 2015; Jarvis et al., 2013). Second, after ART initiation, some patients develop Cryptococcal immune reconstitution inflammatory syndrome, where damage results from an exaggerated Th1 response, marked by increased IFN-γ and IL-6, activation of monocytes/macrophages, and CD4^+^ T-cell recruitment (Pinner et al., 1995). Activated macrophages’ neurotoxic effects, the induction of cerebral edema, and the metabolic programming of neurons by nearby inflammatory signals are a few of the processes of immune-mediated brain injury (Chang et al., 2013; Boulware et al., 2010). Although normal Th1 signaling was detected in the cerebrospinal fluid (CSF) of the aforementioned cohort of non-HIV patients with cryptococcal meningitis, autopsy analyses revealed a predominance of alternatively activated (M2) macrophages within central nervous system (CNS) tissues (Panackal et al., 2015; Olszewski et al., 2010). A similar immunologic pattern has been observed in patients harboring autoantibodies against granulocyte–macrophage colony-stimulating factor (GM-CSF), who are likewise susceptible to the infection. Despite exhibiting abundant Th1 CD4^+^ T-cell responses, these patients demonstrate a skewing toward an M2 macrophage phenotype, suggesting impaired effector macrophage function (Saijo et al., 2014; Panackal et al., 2016).

In this review, we focus on the immunopathology associated with cryptococcal disease, as well as the challenges and recent advances in addressing this global health issue. Given the high mortality and limited therapeutic options, there is a pressing need for a comprehensive understanding of this disease. We highlight the adaptive and intrinsic mechanisms used by *Cryptococcus* species to promote immune evasion and drug resistance, and discuss progress in developing new antifungal small molecules as potential therapeutic strategies.

## 2 Immunopathology in cryptococcal infections

Several physical characteristics and anatomical barriers play a crucial role in preventing *C. neoformans* from establishing infection in mammalian hosts, even though these defences are sometimes overlooked.

The skin serves as the first line of defense against fungal pathogens. Its intact epidermal layer provides an effective mechanical and immunological barrier, making direct penetration by *C. neoformans* highly unlikely under normal conditions. In contrast, the upper respiratory tract and nasal airway openings represent significant potential portals of entry. Because inhalation is the primary route of exposure, fungal cells can bypass the protective epidermis and enter through the respiratory mucosa. Kuttin et al. (1988) demonstrated that *C. neoformans* is capable of penetrating the mucosal and nasal epithelial layers in mice and rats, highlighting the vulnerability of these tissues. The anatomical connection between the nasal cavity and the subcranial region further suggests a possible pathway for fungal dissemination to the CNS. This relationship raises the possibility that, after colonizing the nasal passages, *C. neoformans* may access the brain either through local tissue invasion or via neural or vascular routes. Overall, while the skin provides strong protection, the respiratory and nasal mucosa represent critical sites that may allow *C. neoformans* to overcome host physical barriers and initiate infection. Cryptococci can reach the CNS from different sites via multiple pathways. In rare cases like cryptococcal laryngitis, they accumulate in the throat, enter the bloodstream, and cross the blood–brain barrier to the brain. It is widely accepted that humans primarily acquire pulmonary infection with *C*. *neoformans* through inhalation of airborne basidiospores or desiccated yeast particles (Velagapudi et al., 2009). Once inhaled, these organisms deposit in the respiratory tract and may establish infection in the lungs before potentially disseminating to other organs. Spore-infected mice show a higher brain fungal burden than yeast-infected mice, likely due to events in the lungs before bloodstream spread (Walsh et al., 2019; Frerichs et al., 2021). However, both spores and yeast cross the blood-brain barrier (BBB) by similar mechanisms. Spores may germinate into yeast within macrophages and disseminate to the brain, possibly also spreading directly from sinus infections (Frerichs et al., 2021; Giles et al., 2009). Although cryptococcal choroid plexitis suggests a possible entry route into cerebrospinal fluid via the choroid plexus, studies have not observed yeast cells or structural damage in this region in mouse models (Dubbioso et al., 2013; Charlier et al., 2005; Chang et al., 2004).

Interestingly, temperature plays an important role in fungal survival and growth. *Cryptococcus neoformans* demonstrates optimal growth at environmental temperatures of approximately 25 °C–30 °C. In contrast, its growth efficiency decreases at the human core body temperature of 37 °C. This thermal restriction represents a physiological barrier that many environmental fungi cannot overcome (Kuhn, 1939). With the exception of the tiny basidiospores, ciliary activity and airway turbulence are typically effective in keeping yeast cells from entering the alveoli. An overreactive inflammatory response and/or direct host injury can be caused by invasion of the bronchial epithelium alone (Casadevall et al., 1998). *Cryptococcus neoformans* var. *Gatti*, for example, has been linked to the aggressive development of granulomatous masses in the lungs (Chen et al., 2000). *Cryptococcus neoformans* meningoencephalitis, a symptomatic infection that usually results in death if left untreated, can be caused by a more serious systemic infection, which is primarily observed in immunocompromised patients.

Macrophages play a central role in host defense against *Cryptococcus* species by mediating capsule polysaccharide recognition, phagocytosis, intracellular killing, cytokine and chemokine secretion, and antigen presentation (Barreto-Bergt et al., 2014; Trevijano-Contador et al., 2018). Traditionally, cryptococcal phagocytosis has been considered largely opsonin-dependent, requiring antibody- and complement-mediated mechanisms. In particular, antibodies targeting the capsular polysaccharide glucuronoxylomannan (GXM) bind through their Fc regions to macrophage Fc receptors, while activation of the C3–C3b complement pathway promotes complement receptor–mediated uptake (Rohatgi and Pirofski, 2015). Together, these pathways facilitate efficient internalization of the pathogen.

However, accumulating evidence indicates that macrophages can also internalize *Cryptococcus* through non-opsonic mechanisms involving direct recognition of fungal pathogen-associated molecular patterns (PAMPs) by pattern recognition receptors (PRRs) (Lim et al., 2018). Unlike the classic mannoprotein–mannose receptor interaction observed in many other fungi, recognition of *Cryptococcus* predominantly relies on spleen tyrosine kinase (Syk)-dependent signaling pathways. Specifically, *C. neoformans* can be recognized by both Dectin-1 and Dectin-2 receptors, whereas *C. gattii* is primarily detected by Dectin-1. These differences reflect species-specific variations in cell wall PAMP composition (Lim et al., 2018).

In mammalian hosts, *Cryptococcus* sp. Expresses a number of virulence factors that promote pathogen survival, growth, and spread (Alspaugh, 2015; Diaz, 2020). Proteases, ureases, phospholipases, and nucleases are among the degradative enzymes that *C. neoformans* creates at the molecular level to break down host molecules (Almeida et al., 2015; Hamed et al., 2023). The mechanisms of cellular damage include disruption of phagolysosome maturation (Smith et al., 2015), increased permeability of the phagosome membrane (DeLeon-Rodriguez and Casadevall, 2016), disruption of organelle function, such as the reduction of mitochondrial protein synthesis (Coelho et al., 2015), cytoskeletal changes (Ki et al., 2012), non-lytic exocytosis and lytic exocytosis leading to host cell death (Stukes et al., 2016; O’Meara et al., 2015). Mitochondria have long been linked to drug resistance in fungi. They are linked to the endoplasmic reticulum (ER) by a multiprotein complex called the ER-mitochondria encounter structure (ERMES), which is unique in the fungal kingdom. In an investigation on *C. neoformans*, the four ERMES complex subunits were removed to create mutant strains. These mutants exhibited defective mitochondria and were vulnerable to antifungals, particularly echinocandins, due to their low chitin level (Kumari et al., 2025). The mutants lacked virulence factors like as capsule formation and melanin synthesis. The disrupted ERMES interaction impaired the activity of numerous ER-synthesized enzymes implicated in virulence. *In vivo* experiments in the *Caenorhabditis* worm model of cryptococcosis verified the mutants’ lower pathogenicity.

Owing to the special traits of *Cryptococcus* sp., cryptococcal infections are very difficult to treat in contrast to other systemic fungal diseases. *Cryptococcus sp.* has developed a number of distinct tactics that help it persist and survive in the host without showing signs of illness. *Cryptococcus* sp. is primarily an environmental yeast and humans are considered accidental hosts. Therefore, the driving forces behind its virulence traits come from environmental survival pressures, not human infection. Specifically, features like the polysaccharide capsule and the ability to survive inside phagocytic cells help it persist in nature. These same adaptations coincidentally enable it to evade the human immune system. Interestingly, the development of a protective cell-mediated immune response upon secondary infection is unaffected by the persistence of a chronic, low-grade *C. neoformans* infection (Lindell et al., 2006). The ability of environmental cryptococci to adapt to a wide range of environments is demonstrated by the fact that they may infect a large number of vertebrate and invertebrate hosts (Shourian and Qureshi, 2019). Fungal survival in the mammalian host is mostly dependent on metabolic adaptation, and numerous genes and pathways necessary for stress tolerance and growth at high temperatures have been found (Rosa e Silva et al., 2008). Ras1/Ras2 signaling pathways and functional calcineurin A, which is triggered by stress responses and promotes the expression of genes necessary for growth and survival at 37 °C and oxidative stress conditions (Maeng et al., 2010; Kraus et al., 2005).

**FIGURE 1:**
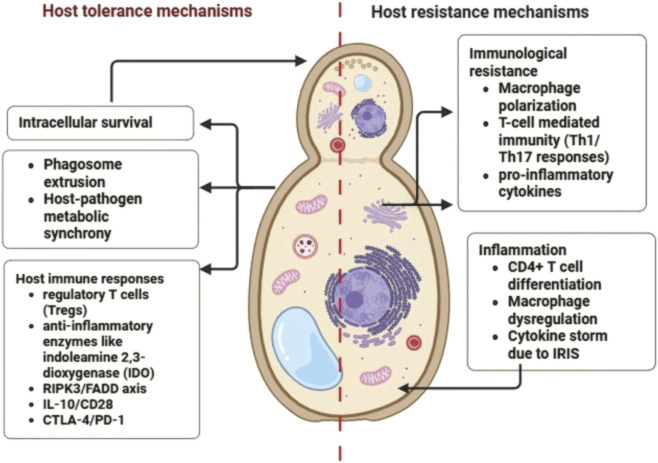
This schematic illustrates the balance between resistance and tolerance mechanisms in cryptococcal infection.

Numerous factors that have been demonstrated to disrupt the host immune response are expressed by *Cryptococcus* sp. (Figure 1). Cell wall antigens are hidden by the capsule, which also prevents antibodies from attaching to the fungal cell wall, activates and depletes complement, reduces T lymphocyte proliferation, alters cytokine production, and triggers host cell death (Zaragoza et al., 2009). *Cryptococcus sp.* employs a potent anti-phagocytic strategy that involves capsular growth during infection and the development of enormous “Titan cells” that range in size from 50 to 100 µm (Okagaki and Nielsen, 2012). When capsular glucuronoxylomannan is released, neutrophils shed L-selectin, which restricts their ability to migrate, adhere to endothelial cells, and extravasate into tissue (Casadevall et al., 2018). Additionally, the anti-inflammatory qualities of cryptococcal capsular components prevent dendritic cells (DCs), macrophages, and neutrophils from maturing and activating (Kelly et al., 2005; Monari et al., 2002). Neutrophil migration and the formation of neutrophil extracellular traps (NETs) (Rocha et al., 2015) are both inhibited by capsular and cell wall components of *C. neoformans* (Coenjaerts et al., 2001; Ellerbroek et al., 2004). In addition, melanized *C. neoformans* cells prevent neutrophil death by blocking sphingomyelin synthase (SMS) activity, a process required for neutrophil-mediated killing of the fungus (Qureshi et al., 2010; Qureshi et al., 2011). By secreting capsular components that stimulate microglia to produce IL-8, a potent neutrophil chemoattractant, cryptococcal cells paradoxically impair neutrophil recruitment. In humans with disseminated cryptococcosis, these capsular products are associated with decreased expression of L-selectin (CD62L) on the neutrophil surface, thereby reducing neutrophil migration. Additionally, cryptococcal capsular polysaccharide impairs TNF-α receptor surface expression and disrupts neutrophil endothelial rolling (Urban et al., 2006). *Cryptococcus sp.* expresses a number of enzymes involved in nitric oxide detoxification and oxidative damage repair, including catalases, superoxide dismutases, glutathione peroxidases, thioredoxin proteins, the protein kinase C (Pkc1), the isositol phosphatidylglycerol-phospholipase C1 (Isc1), and the protein kinase C (Pkc1), in order to survive in the harsh phagosomal environment. They also use host lipid components to produce cryptococcal eicosanoids (Seider et al., 2010). Phospholipase (PLB1), urease, melanin, laccase, and heat shock protein 70 homolog Ssa1 are additional elements that support intracellular survival and persistence (Lange et al., 2023; Zhu and Williamson, 2004; Eastman J. et al., 2015). Evidence demonstrates that *C. neoformans* can disseminate to the central nervous system (CNS) by employing a Trojan horse mechanism, whereby it survives within macrophages in the bloodstream to cross the blood–brain barrier. After entering the CNS, the organism is released from macrophages through a process of non-lytic extrusion (Ma et al., 2006; Zhang et al., 2015; Sorrell et al., 2016; Nicola et al., 2011).

*Cryptococcus sp.* regulates T-cell survival, differentiation, and proliferation through several pathways (Mukaremera and Nielsen, 2017; Almeida et al., 2001). For example, cryptococcal urease attracts immature DCs to lung-associated lymph nodes, eliciting a non-protective and detrimental Th2 response (Osterholzer et al., 2009a). In pulmonary infection, host chiotriosidase-induced cleavage of fungal chitin triggers Th2 cell development by CD11b+ dendritic cells (Wiesner et al., 2015). *Cryptococcus neoformans* production of PGE2 selectively suppresses IL-17 expression during Th17 development in an IRF4-dependent manner (Valdez et al., 2012). Inhibiting the Th17 response may promote latent infection (Olszewski et al., 2010). Chronic lung *C. neoformans* infection also specifically reduces antibody responses to exogenous cryptococcal polysaccharide, impairing humoral immunity (Goldman et al., 2000). Host resistance features include Th1/Th17 responses, inflammatory DC recruitment, pro-inflammatory cytokine release, and classical macrophage activation (Wozniak et al., 2009; Murdock et al., 2014; Valdez et al.; Hoag et al., 1997; Zhang et al., 2009; Kleinschek et al., 2010; Kleinschek et al., 2006). Conversely, excessive inflammation and strong Th1/Th17 responses that achieve sterilizing immunity can cause significant pathology and host damage (Panackal et al., 2015; Fa et al., 2017; Eastman AJ. et al., 2015).

Fungal traits, as well as the expression of cytokines, chemokines, and scavenger receptors locally, influence the recruitment and maturation of DCs and their capacity to activate T cells. In several models of cryptococcal infection, a number of soluble mediators, such as IL-4, IL-10, IL-17, and GMCSF, have been linked to the activation, differentiation, and recruitment of DCs. Recruitment and classical activation of monocyte-derived DCs (moDCs), which result in the release of pro-inflammatory cytokines and efficient Th1/Th17 immune responses, are linked to protection against *C. neoformans* (Osterholzer et al., 2009b). However, moDCS are extremely versatile cells that, depending on the local cytokine microenvironment within infected tissues, can exhibit either inflammatory or immunoregulatory functions (Roussey et al., 2016). The immunomodulatory cytokine IL-10 was significantly generated by human and murine monocytes and DCs activated *in vitro* with *C. neoformans* antigen (Vecchiarelli et al., 1996). Moreover, Th1 and Th17 suppression, decreased macrophage activation, and compromised fungal clearance were linked to the formation of immunomodulatory DCs in a mouse model of chronic *C. neoformans* infection (Chen et al., 2023; Hernandez et al., 2005). A mouse model of chronic lung infection with *C. neoformans* 52D showed early and maintained IL-10 production by lung leukocytes (Chen et al., 2023). When infected with *C. neoformans*, C57BL/6 mice with genetically modified IL-10 deficiency showed enhanced fungal clearance from the lung in conjunction with decreased tissue eosinophilia, along with reduced Th2 cytokines expressions, and upregulated Th1 cytokines by lung leukocytes (Hernandez et al., 2005).

Mutations in the Treg-associated transcription factor forkhead box protein P3 (FOXP3) are linked to severe immunopathology in both humans and animals. This suggests that Tregs regulate tissue damage and help control disease (Soares et al., 2017). The activation of Treg cells is crucial to restoring a balanced environment and reducing tissue damage during fungal infection (Roussey et al., 2016). Treg function is associated with the production of anti-inflammatory cytokines TGF-β and IL-10 (Roussey et al., 2016; Romani et al., 2011). In BALB/c mice, peripheral CD4^+^ FoxP3+ Tregs increased during the first 4 weeks of *C. neoformans* 1841 infection. When Tregs were conditionally depleted in the second week (while Th1 and Th2 responses were ongoing), the Th2 response increased. This suggests Tregs limit Th2 cell proliferation and activity (Romani et al., 2011). Other research showed CCR5 and IFN regulatory factor 4 (IRF4) boost formation of antigen-specific Tregs in *Cryptococcus*-infected lungs and their co-localization with Th2 cells (Wie et al., 2016). The immunosuppressive activity of Tregs during early cryptococcal infection correlates with decreased harmful Th2-mediated immune responses; however, whether prolonged cryptococcal infection is likewise linked to enhanced Treg activity remains unclear and requires further investigation (Olszewski et al., 2010; Roussey et al., 2016). *Cryptococcus neoformans* quickly leads murine CD4^+^ T cells to upregulate CTLA-4 (Pietrella et al., 2001). Blocking CTLA-4 caused these CD4^+^ T cells, activated by *C. neoformans*, to produce more IL-2/IFN-γ and proliferate more. The use of encapsulated *C. neoformans* strains led to different CTLA-4 expression patterns. Blocking CTLA-4 also improved the survival and fungal control in mice with highly virulent *C. neoformans* virus (McGaha and Murphy, 2000). These results suggest that inducing CTLA-4 helps cryptococci dampen the immune response and promote persistent infection (Roussey et al., 2016). Another study demonstrated that blocking CTLA-4–mediated signaling during immunization with cryptococcal antigens or during active infection leads to enhanced cell-mediated immunity (CMI), as evidenced by increased anticryptococcal delayed-type hypersensitivity (DTH) responses (Nichols et al., 2002). It is widely recognized that engagement of CTLA-4 with B7 ligands suppresses T-cell activation by inhibiting the production of the growth factor IL-2 and downregulating expression of the IL-2 receptor α-chain, which is essential for IL-2 signaling (Krummel and Allison, 1996; Walunas et al., 1996). Additionally, CTLA-4 ligation interferes with cell cycle progression by preventing activation of key regulatory proteins, including cyclin D3 and cyclin-dependent kinases 4 and 6 (Krummel and Allison, 1996; Walunas et al., 1996; Brunner et al., 1999; Blair et al., 1998).

## 3 Antifungal resistance mechanisms

**FIGURE 2:**
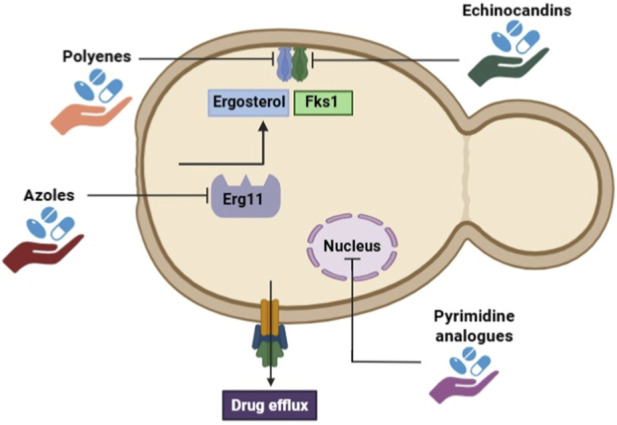
Mechanisms regulating resistance to current antifungal drugs.

The only antifungal medications now available for treating invasive fungal infections are polyenes, azoles, and echinocandins, notwithstanding the concerning effects of these infectious agents on human health (Table 1 and Figure 2) (Lee et al., 2021). While the azoles directly prevent ergosterol manufacture by blocking the activity of lanosterol 14α-demethylase, the polyenes target and deplete the critical membrane lipid ergosterol from the plasma membrane. By preventing the synthesis of the essential cell wall component (1,3)-β-d-glucan (Lee et al., 2021), the echinocandins compromise the integrity of fungal cell walls. Last but not least, the pyrimidine analogue flucytosine, sometimes referred to as 5-fluorocytosine, works as an antimetabolite that eventually stops DNA synthesis; nevertheless, its use as a monotherapy is prohibited due to the frequent development of resistance. Regrettably, drug resistance to all three types of antifungal drugs has rapidly emerged as a result of broad antifungal use (Fisher et al., 2018).

**TABLE 1:** Summary of available therapeutic compounds against the pathogenesis against *Cryptococcus* spp.

Compounds	Mechanism of action	References
Benzothioureas	Suppress the post-golgi (late secretory pathway), potentially by directly interacting with Sav1, an orthologue of Sec4-class small GTPase	Beattie et al. (2020)
Clofazimine	Induces a cell membrane stress	Iroh Tam et al. (2021)
Polyene antibiotics	Ergosterol-specific and inhibition of membrane transport proteins	te Wel et al. (2012)
Pyrimidine analogues	Inhibits DNA and RNA synthesis	Basha and Goudgaon (2021)
Azoles	Targets enzyme lanosterol 14-α-demethylase	Carvajal et al. (2023)
N′-(3-bromo-4-hydroxybenzylidene)-2-methylbenzohydrazide (BHBM) and its derivative, 3-bromo-n′-(3-bromo-4-hydroxybenzylidene) benzohydrazide (D0)	Targets sphingolipid glucosylceramide (GlcCer)	Mor et al. (2015)
Macrolactone, ibomycin	Targets cell membrane	Robbins et al. (2016)
Resorcylate aminopyrazoles	Hsp90 inhibitor	Marcyk et al. (2021)
VT-1598 and oteseconazole (VT-1161)	CYP51 inhibitor	Wiederhold et al. (2018), Gu et al. (2024)
Tamoxifen	Repurposed drug that targets calmodulin	Dolan et al. (2009)
Fosmanogepix	Targets fungal glycosylphosphatidylinositol-anchored cell wall transfer protein 1	Shaw et al. (2018), Hodges et al. (2023)
Sertraline	Targets ergosterol biosynthesis	Gowri et al. (2020)

Due to *Cryptococcus* species’ inherent resistance to β-d-glucan inhibitors and their use of a variety of defense mechanisms that allow for resistance to azoles treatment options for invasive cryptococcal infections are restricted (Denning, 2003). Therefore, for over 50 years, the polyene amphotericin B has been the main treatment for cryptococcal infections, even though it is highly toxic and its availability is restricted by logistical and economic factors (Perfect et al., 2010). Liposome bilayer-coated amphotericin B (LAmB) has been developed to lessen toxicity while maintaining antifungal activity, thereby reducing the dosage limitation of conventional amphotericin B deoxycholate (DAmB) (Stone et al., 2016). These lipid formulations exhibit such promise that a single high dose of LAmB is as effective as a 7-day course of DAmB (Jarvis et al., 2019). Amphotericin B is encapsulated in a lipid-containing crystal nanoparticle in a novel form of encochleated oral amphotericin B (CAmB), which is efficiently transported to the central nervous system and has antifungal activity in a mouse model (Lu et al., 2019). Clinical studies for CAmB are under underway, and the outcomes will be particularly significant because this formulation will eliminate the need for expensive intravenous delivery of existing medication forms (US National Library of Medicine, 2019). In particular, amphotericin B plus the fluorinated pyrimidine analogue flucytosine, followed by the triazole fluconazole, increased survival rates and reduced toxic effects when compared to amphotericin B alone in a clinical trial involving HIV + patients with cryptococcal meningitis (Day et al., 2013).

The main mechanism by which *Cryptococcus* can modify its genomic structure in response to antifungal stress is known as heteroresistance. Heteroresistance is a significant therapeutic concern because it contributes significantly to relapse during azole maintenance therapy (Stone et al., 2019; Rhodes et al., 2017) since the percentage of resistant cells rises with treatment. *In vitro* and *in vivo*, heteroresistance mostly happens through the production of aneuploid cells (Sionov et al., 2010; Sionov et al., 2013), most frequently through chromosome one disomy, which contains *ERG11* and the dominant efflux pump gene *AFR1* (Sionov et al., 2010). Chromosome four disomy is the second most prevalent aneuploidy that results in azole heteroresistance, and it is mostly caused by three genes: *GLO3* and *GCS2*, which encode ADP-ribosylation factor proteins, and *SEY1*, which encodes a GTPase. Other chromosomes, such as chromosomes 6, 10, 11, and 14, have also been observed to be aneuploid, although it is still unknown which particular genetic components provide the selective advantage (Ngamskulrungroj et al., 2012; Gerstein et al., 2015). Lastly, while the exact mechanism behind the generation of aneuploidy in *Cryptococcus* sp. Is still unknown, evidence indicates that aneuploid cells under azole stress are produced either by chromosome mis-segregation or by impaired nuclear division (Altamirano et al., 2017; Chang et al., 2018). Recent research on the mutations in *C. gattii* that cause resistance to flucytosine revealed that, in comparison to isolates without a hypermutator mutation, hypermutator strains have higher resistance to fluorouracil. The genes encoding the purine-cytosine permease *Fcy2* and the uracil phosphoribosyltransferase *Fur1*, and the gene encoding *Uxs1*, which generates UDP-xylose, a precursor in capsule biosynthesis (Billmyre et al., 2020), were mutated to cause this increased resistance.

Resistance and virulence are also influenced by modifications to the *Cryptococcus* capsule and cell wall. For instance, *C. neoformans* cells with larger capsules are more virulent and resistant to amphotericin B (Park et al., 2014), and older *C. neoformans* cells’ thicker cell walls have been linked to their higher resistance to antifungals (Bouklas et al., 2013). Antiphagocytic protein one and the laccase enzymes Lac1 and Lac2 are established virulence factors of *C. neoformans*, contributing to pathogenicity by inhibiting phagocytic uptake and promoting melanization (Qureshi et al., 2012). One study demonstrated that older cells exhibit greater levels of melanization than younger cells and, consequently, show increased resistance to amphotericin B–mediated killing (Orner et al., 2019). Reduced melanization in aged lacΔ mutants was associated with diminished resilience, indicating that age-dependent melanization, mediated by laccase genes, enhances the overall robustness of older cells (Orner et al., 2019). Furthermore, older cells were found to be more resistant to macrophage phagocytosis; however, this resistance was abolished when antiphagocytic protein one was deleted. This finding suggests that upregulation of antiphagocytic protein one in aged cells partially accounts for their enhanced resistance to macrophage-mediated phagocytosis. Lastly, the development of *Cryptococcus* titan cells, which show several changes that probably lead to drug resistance, is an example of the ability of fungi to go through drastic morphological changes (Zafar et al., 2019).

One of the main obstacles to treating cryptococcosis is the innate resistance of *Cryptococcus* sp. To echinocandins. This resistance is contradictory because echinocandins’ enzymatic target, β-1,3-glucan synthase, is crucial for *Cryptococcus* and has been shown to be strongly biochemically inhibited by them (Thompson et al., 1999; Maligie and Selitrennikoff, 2005). Cell division cycle 50 (CDc50), which codes for the β-subunit of a lipid lipase enzyme, was found to be essential for echinocandin resistance through a genetic screen of *C. neoformans* mutants. It was also shown to play a role in preserving the phospholipid membrane’s lipid asymmetry (Huang et al., 2016). Furthermore, a CDc50 mutant treated with capsafungin showed increased intracellular calcium and hyperactivation of calcineurin-dependent stress responses (Cao et al., 2019). Cdc50 has a direct interaction with Crm1 and inhibits its expression (Cao et al., 2019). Accordingly, it was hypothesized that calcineurin signaling (via Crm1) mediates echinocandin resistance in *Cryptococcus* and is overstimulated due to CDc50 deletion, ultimately leading to cell death (Cao et al., 2019).

Cdc50-deficient mutants show increased sensitivity to caspofungin, prompting the development of an antifungal peptide targeting P4-ATPase (Tancer et al., 2022). Stable Cdc50 loop-derived peptides were screened, and a myristylated AS15-based peptide showed activity at higher doses. An optimized variant, AW9-Ma, had a MIC of 64 μg/mL against wild-type *C. neoformans* and showed synergy with caspofungin (Tancer et al., 2022). In combination, it reduced caspofungin MIC to 4 μg/mL, matching the sensitivity of the *cdc50Δ* mutant. Flow cytometry confirmed flippase inhibition and phosphatidylserine accumulation on the cell surface. Fluorescent imaging showed co-localization with P4-ATPase Apt1, and structure–activity analysis identified two key lysine residues essential for activity. Cryptomycinamide (KKOO-NH2) is an antifungal peptide derived from a 9–amino acid segment of the *C. neoformans* Cdc50 protein (Tancer et al., 2024). It shows broad antifungal effects against several major pathogens such as *C. neoformans*, *Candida glabrata*, *Candida albicans*, *Candida auris*, and *Aspergillus fumigatus*. The peptide has low host-cell toxicity and exhibits strong synergy with amphotericin B, itraconazole, and caspofungin. It localizes to the fungal plasma membrane, likely disrupting Cdc50–P4 ATPase interactions, and induces oxidative stress and calcium influx in fungal cells. It also reduces intracellular proliferation of *Cryptococcus* within macrophages, suggesting potential therapeutic relevance. Butyrolactol A, identified through a natural product screen, restores caspofungin activity against *C. neoformans* H99 and enhances echinocandin efficacy against resistant fungi, including *C. auris* (Chen et al., 2026). It inhibits the phospholipid flippase Apt1–Cdc50, trapping it in an inactive state and disrupting membrane asymmetry, vesicular trafficking, and cytoskeletal organization. This increases drug uptake and potency, highlighting lipid flippases as promising antifungal targets and supporting the potential of natural products to combat resistance (Chen et al., 2026).

In addition, fungal biofilms may form on implanted medical devices, such as catheters, serving as reservoirs from which fungal cells can disseminate into the bloodstream and spread to distant organs, potentially leading to severe and life-threatening invasive infections (Tits et al., 2020; Silva et al., 2017). A promising strategy to overcome the limited range of available antibiofilm therapies involves combining an antifungal agent with a non-antifungal potentiator to improve its antibiofilm efficacy. There is a notable shortage of combination therapies targeting biofilms formed by *Cryptococcus* species. Nevertheless, the biofilm state plays a critical role in *C. neoformans* infection, contributing to its survival within macrophages, facilitating invasion of the central nervous system, and promoting colonization of host tissues (Aslanyan et al., 2017).

## 4 Potential therapeutic small molecules

The majority of compound libraries have been created to maximize their “drug-like” qualities with regard to mammalian targets and physiology, while antifungal drugs require special physicochemical properties, partly because these molecules must be able to pass through the fungal cell wall. As a result, the development of new antifungal molecules continues to be a significant challenge. Dark chemical matter presents a potentially exciting source of new antifungal treatments because these compounds exhibit no biological activity when examined in various screening programs. These seemingly inert substances are not biologically inert, according to a thorough analysis that also found a strong inhibitor of *C. neoformans* development *in vitro* (Wassermann et al., 2015). Additionally, due to the distinct neurological characteristics of cryptococcal illness, antifungal compounds with strong central nervous system penetration and oral administration capabilities are required to provide broad access in developing nations with constrained resources. This insight has prompted the use of non-traditional chemical libraries in antifungal screening initiatives.

New articles highlight promising antifungal leads for optimization. However, the mechanisms behind their activity are often not fully understood, and potential challenges from certain chemical features may be overlooked. Research suggests that many of these leads could be non-optimizable due to the presence of pan assay interference compounds (PAINS) or other promiscuous groups (Pouliot and Jeanmart, 2016). By recognizing these issues, we can better focus on more viable candidates for development. Baell and Holloway (2010) released his findings on PAINS, which includes a compilation of structural characteristics of common false positives from six distinct and independent assays. Although PAINS were recognized through target-based high-throughput screening (HTS), they also hold significant importance for the hits from phenotypic screening. All the compounds showed promising antifungal activity in initial screenings. However, when evaluated in higher-tier assays, none were able to demonstrate significant effectiveness. This suggests that while these compounds may impact fungal targets, they may also be interacting with non-target organisms in a non-selective way.

Additionally, by screening a wide range of strains of actinomycetes that produce natural products, the glycosylated macrolactone ibomycin was found to be an inhibitor of *C. neoformans* growth because it can penetrate the fungal cell wall and alter membrane function by interfering with endosomal trafficking (Robbins et al., 2016). Aside from using unconventional chemical libraries to find new antifungals, other high-throughput screening tools that can detect fungicidal compounds specifically have been created (Hartland et al., 2016; Rabjohns et al., 2014). These platforms have been crucial in locating new pharmacological targets and effective compounds, especially when used in conjunction with proteomic and genomic techniques to reveal the targets of compounds (Park et al., 2016; Xue et al., 2021).

A recent study developed a novel on-resin macrocyclization strategy to design inhibitors of Major aspartyl peptidase 1 (May1), a secreted protease essential for low-pH survival and virulence in *C. neoformans* (Kryštůfek et al., 2025). May1 is a member of the peptidase A1 family and shares strong structural similarity with other fungal secreted aspartyl proteases, including SAPs from *C. albicans* (Kryštůfek et al., 2021). In a mouse infection model, deletion of the may1 gene (may1Δ) resulted in markedly reduced pathogenicity, extending host survival by more than twofold (Clarke et al., 2016). Using a range of aliphatic linkers and statine-based transition-state mimetics, they synthesized a focused library of 624 macrocyclic compounds. Screening identified several subnanomolar inhibitors with favorable pharmacokinetic and antifungal properties. Notably, lead compound 25 showed a Ki of 180 pM, high selectivity against host proteases, and strong antifungal activity *in vitro*.

An important and intricate organelle that preserves cellular integrity and allows the fungus to interact with its surroundings is the fungal cell wall. Since mammalian cells lack this structure, significant efforts have been made to create antifungal drugs that either directly target this special structure or prevent its formation. To find agents with fungicidal activity against *C. neoformans*, a high-throughput screen of over 300,000 compounds from the US National Institutes of Health (NIH) Molecular Libraries Program was conducted. Secondary assays gave priority to compounds that disrupted the integrity of the fungal cell wall (Hartland et al., 2016).

N-substituted benzothioureas were identified as a scaffold that can prevent *Cryptococcus* sp. From activating the cell wall integrity pathway (Hartland et al., 2016). These molecules were specifically found to be direct inhibitors of the Sec4-class small GTPase Sav1, which impairs the late post-Golgi secretory pathway and reveals a novel method of compromising cell wall integrity and preventing fungal cell growth (Beattie et al., 2020). A promising target for antifungal therapy is Gwt1, an essential virulence factor and the enzyme that catalyzes an early stage in glycosylphosphatidylinositol (GPI)-anchor biosynthesis (Zha et al., 2015). The Gwt1 inhibitor APX001, commonly known as Fosmanogepix, the first clinical candidate in the gepix structural class of molecules, has been effective against *Cryptococcus* spp. (Shaw et al., 2018) both *in vitro* and *in vivo*. A mouse model of cryptococcal infection (Rittershaus et al., 2006) showed that APX001, either by itself or in conjunction with fluconazole, dramatically reduced the fungal burden in the brain and lungs. Phase II trials for the treatment of invasive infections brought on by *Aspergillus* and *Candida* species are presently in progress (NCT02957929, NCT02956499, and NCT04240886), underscoring the potential broad-spectrum utility of this drug candidate. Clinical trials using APX001 for the treatment of cryptococcosis are set to begin shortly. Lastly, because sphingolipids have a role in modulating signal transduction, cell control, and virulence in fungal infections, their production is becoming a desirable target (Singh et al., 2012). Two compounds were found to be highly effective antifungal agents in cryptococcosis100 by specifically targeting the synthesis of the fungal sphingolipid glucosylceramide (GluCer): N'-(3- bromo- 4-hydroxybenzylidene)-2-methylbenzohydrazide (BHBM) and its derivative 3-bromo- N'-(3- bromo- 4-hydroxybenzylidene)benzohydrazide (B0).

Antifungal compounds with enhanced efficacy and fungal selectivity have been created to capitalize on known targets, such as Erg11 (sometimes referred to as Cyp51), in addition to the creation of medications with new targets. When compared to existing authorized Erg11 inhibitors, such as fluconazole (Garvey et al., 2018), the tetrazole VT-1598 is a logically constructed Erg11 inhibitor with improved fungal selectivity. VT-1598 exhibits broad-spectrum efficacy against a variety of fungus, including a good therapeutic promise in a rat model of cryptococcal meningitis (Garvey et al., 2018), much like other clinically licensed azoles.

It is possible to repurpose compounds created for other therapeutic purposes if it turns out that they also have antifungal capabilities, which would speed up the creation of new antifungal medicines. One class of anthelmintic drugs that has been used in clinical settings for many years is benzimidazoles (McKellar and Scott, 1990). In a mouse model of cryptococcosis, fenbendazole exhibits strong *in vivo* efficacy against *C. gattii* and *C. neoformans* (de Oli et al., 2020). The growth-inhibiting qualities of fenbendazole, its suppression of *Cryptococcus* virulence factors, and its suppression of fungal proliferation within macrophages were credited with its effectiveness (de Oli et al., 2020). Given that both tamoxifen and sertraline made it to clinical trials, these medications are arguably the most prominent examples of chemicals being repurposed to treat cryptococcosis. Sertraline, which was first created as an antidepressant, works in concert with fluconazole to lower the fungal burden in a mouse model of systemic cryptococcosis (Zhai et al., 2012).

Regretfully, sertraline medication, either by itself or in conjunction with amphotericin B and diflucan, did not enhance patient outcomes in a phase III trial with patients receiving treatment for cryptococcal meningitis (Rhein et al., 2016), despite encouraging results in phase I/II clinical trials. For the treatment of cryptococcal meningitis (NCT03112031), a phase II clinical trial evaluating the safety and effectiveness of tamoxifen (300 mg daily) as an adjuvant therapy to the standard therapy of amphotericin B and fluconazole has concluded (Ngan et al., 2019); however, the trial did not find any therapeutic benefit of this combination when compared to the standard therapy alone. Although sertraline and tamoxifen showed antifungal activity in preclinical and early-phase studies, the effective concentrations may not be safely achievable in humans, particularly within the CNS where *C*. *neoformans* persists. Limited penetration into cerebrospinal fluid, along with metabolism and protein binding, further reduces active drug levels at the site of infection. In addition, their antifungal effects are relatively weak compared to standard fungicidal therapy, making them insufficient for the rapid fungal clearance required in cryptococcal meningitis. Outcomes are further influenced by severe immunosuppression in patients with HIV, potential drug interactions with standard treatments, and the broader heterogeneity of patients enrolled in phase III trials, which often diminishes signals of benefit seen in earlier studies.

A major unmet need in the treatment of cryptococcal meningitis is the lack of effective antifungal agents with reliable central nervous system (CNS) penetration. Many currently available or repurposed drugs have limited ability to cross the blood–brain barrier, resulting in subtherapeutic concentrations in the cerebrospinal fluid. In addition, some agents that do reach the CNS fail to maintain sustained fungicidal activity, are constrained by dose-limiting toxicity, or are significantly affected by protein binding and host metabolism. These limitations collectively reduce treatment efficacy against *C*. *neoformans* in the brain and underscore the urgent need for novel antifungals specifically optimized for CNS delivery and durable activity at the site of infection.

In addition to finding interesting lead compounds, screening of clinically approved medications also showed patterns in the classes of molecules active against *Cryptococcus* sp. For instance, 31 compounds having fungicidal activity were found via a screen using the Prattwick Chemical Library, which contains 1,120 off-patent medications and compounds (Butts et al., 2013). Antipsychotic medicines constituted the largest class of inhibitors among these 31 compounds, closely followed by general antiseptic agents (Butts et al., 2013). Sertraline, trifluoperazine, and thioridazine are just a few of the antipsychotic medications that were discovered in later research to find chemicals that enhance the activity of fluconazole and amphotericin B against *Cryptococcus* sp. (Spitzer et al., 2011; Rossi et al., 2020). Clofazimine was found to be a broad-spectrum adjuvant therapy that can work in concert with both capsafungin and fluconazole to combat *C. neoformans* and other fungus species in a study evaluating the combination of recognized antifungals with about 3,600 bioactive compounds (Robbins et al., 2015).

## 5 Conclusion

There has never been a more pressing need to fortify the pipeline for developing antifungal drugs due to the ongoing threat that *Cryptococcus* infections pose to public health. The growing interest in novel antifungal mechanisms, such as focusing on vital genes or cellular processes, blocking virulence factors, preventing stress-reaction signaling, using compound combinations, and creating immunomodulatory treatments, gives potential therapeutic options. These incentives, along with developments in genomics technologies, structure-guided drug design, and expanding screening to include structurally diverse compound libraries, will surely lead to important discoveries that will benefit the people who are infected with *Cryptococcus* worldwide.
